# Supplementary material for: Motility of an autonomous protein-based artificial motor that operates via a burnt-bridge principle
Source: Nat Commun. 2024 Feb 23;15:1511. doi: 10.1038/s41467-024-45570-y (PMC10891099; doi:10.1038/s41467-024-45570-y)
Supplement: Supplementary file 1 — Supplementary Information [file 41467_2024_45570_MOESM1_ESM.pdf]

Supplementary Materials for

Motility of an autonomous protein-based artificial motor that operates via a burnt-bridge principle

Chapin S. Korosec[†,*], Ivan N. Unksov[†], Pradheebha Surendiran, Roman Lyttleton, Paul M.G. Curmi, Christopher N. Angstmann, Ralf Eichhorn, Heiner Linke*, Nancy R. Forde*

[†]Equal contributions
*Correspondence to: chapinskorosec@gmail.com, heiner.linke@ftf.lth.se, nforde@sfu.ca

This PDF file includes:

Supplementary Notes 1-3
Supplementary Figures 1-16

Other Supplementary Material for this manuscript includes the following:

Supplementary Movies 1-5

Supplementary Text

Supplementary Note 1: Polyvalency estimates

The original LM concept and design was introduced by Kovacic *et al.* (*1*), though its motility was not tested. In their work, the LM consists of a quantum dot hub decorated with trypsin enzymes (Sigma-Aldrich, T1426). Because of its small size, tracking quantum-dot motion requires fluorescence imaging. Its small size also means that it may penetrate polymer brush lawns, where it has been observed to undergo electrostatically mediated adhesion to the surface (*2, 3*). Furthermore, because Kovacic *et al.* determined that the multivalency of the QD LM is only $N \approx 8$ trypsins (*1*), its processivity and mechanochemical performance may be quite low (*4-6*). A micron-sized hub with larger mass and diameter resolves this issue as the effect of gravity becomes non-negligible and the device will not diffuse away from the surface. Thus, for the work presented here we implement the Lawnmower design using a much larger, 2.8 μm-diameter hub (Supplementary Figure 1).

The polyvalency of the microspherical LM was determined experimentally, as described in Methods: Lawnmower polyvalency and shown in Supplementary Figure 1. Because trypsin is a nonprocessive enzyme (*7*), the activity of a LM scales linearly with the number of tethered, active trypsins. From this value, and literature values on the density of the polymer brush lawn, here we determine the number of trypsins and peptides found in the LM footprint.

We assume that the LM can fully indent the polymer brush, such that area of the spherical cap in contact with the lawn is $A = 2\pi rh$ where the bead radius $r = 1.4$ μm and the height of the lawn is given by h. We estimate h = 12 nm by adding the thickness of the F127 polymer brush (~8.5 nm, when deposited on a hydrophobic surface producing a contact angle of ~100 degrees as was the case here (*8, 9*)), the length of the SMCC crosslinker used to couple the trypsin to the bead (8.3 Å) and the diameter of the trypsin (3 nm (*10*)). This gives a footprint of A = 0.1 μm^2. With the experimentally determined trypsin density on the LM of 0.02 nm^{-2}, we find that the spherical cap can accommodate about $2 \cdot 10^3$ trypsins. In turn, the expected peptide density is that of the PEG arms of the brush: 0.1 nm^{-2} (*11*), and thus the number of peptides that can contact the LM footprint is $1 \cdot 10^4$ peptides. These values represent upper limits of trypsin and peptide number, as they have been determined assuming complete compression of the underlying polymer brush, which is unlikely to be the case.

We can compare this experimental value of polyvalency to two constraining values. First, M-270 amine Dynabeads contain an active amine density of ~150 μmol/g of beads, with a dry weight of 30 mg per $2x10^9$ beads (*12, 13*). This gives $1.3x10^9$ NH_2/bead. This number of reactive groups is easily sufficient to accommodate $5x10^5$ trypsin proteases. One can also calculate the maximum possible packing density of trypsins on the bead surface. Treating trypsins as 3 nm diameter spheres and assuming a hexagonally close-packed structure on the surface of the Dynabead (90% surface packing density), one calculates a maximum of $3x10^6$ trypsins/LM. The experimental value falls below – but reasonably close to – this maximum, suggesting these Lawnmowers are close to the maximum possible polyvalency for this hub size.

Supplementary Note 2: Diffusion coefficients
The values of diffusion coefficients determined for LMs on bare 2D lawns are narrowly dispersed (Supplementary Figure S7D), with $\langle D \rangle = 0.056 \pm 0.004\ \mu m^2 \cdot s^{-1}$ ($n = 59$; error is standard deviation among measurements). This value of diffusion coefficient is less than that predicted by the Stokes-Einstein equation for diffusion of a sphere free in solution,

$$D_0 = \frac{k_B T}{6\pi\eta R}, \tag{S1}$$

where T is temperature, η the viscosity of water, and R the particle radius (here, 1.4 µm). Eq. S1 predicts $D_0 = 0.18\ \mu m^2/s$. Boundary conditions (such as a planar surface in our experiments) influence the fluid flow around an object leading to an increase in drag, and therefore a decrease in diffusion coefficient (*14*). It is therefore expected that our diffusion coefficients are lower than predicted by Eq. S1. The diffusion coefficient for a sphere diffusing parallel to a boundary, $D_{\parallel}$, was developed by Faxén and is given by (*15*)

$$D_{\parallel} = D_0\left(1 - \frac{9}{16}\frac{R}{h} + \frac{1}{8}\frac{R^3}{h^3} + \frac{45}{25}\frac{R^4}{h^4} + \mathcal{O}\left(\frac{R^5}{h^5}\right)\right), \tag{S2}$$

where h is the height from the surface. Using Eq. S2 for our beads with $R = 1.4$ µm that are effectively touching the surface, we calculate that $D_{\parallel}$ is expected to be $\sim 1/3$ of the bulk value predicted from Eq. S1. This agrees favorably with the ratio of our measured diffusion coefficient to the predicted bulk value, further supporting our conclusion that LMs are undergoing two-dimensional diffusion on bare lawns.

For LMs in channels, the variance of the parallel displacement distribution provided a similar value of the diffusion coefficient: $D = 0.06\ \mu m^2/s$.

Supplementary Note 3: Kurtosis
A value of kurtosis above 3.0 for the displacement distributions indicates increased probability of large displacements relative to the central portion of the distribution. For LMs in channels, we found this could result from two possible modifications: (i) increased probability weight around $\Delta x = 0$; and (ii) increased probability weight in the tails of the distribution. For LMs, effect (i) results from including immotile segments, which contribute a narrow peak centered at $\Delta x = 0$ (Supplementary Figure 16). Effect (ii) results from motor activity, as seen in our 1D model results. Thus, when removing the effect (i) by excluding immotile regions from trajectories, any findings of kurtosis >3 should indicate motor-associated motility. This was what we found when analyzing trajectories that we truncated once they appeared immotile.

The motile displacement distribution differs significantly from a Gaussian only in its wings (Figure 2E). By contrast, for the immotile-included distribution shown in Supplementary Figure 16, the ratio of experimental:Gaussian probabilities around zero is significantly greater than one (arising from the contribution of the immotile state).

We found the kurtosis of $\kappa > 3$ to be unaffected by sampling interval. We repeated the analysis of these same motile-only truncated trajectories that had been sampled with $\tau = 1$ s, using longer sampling intervals of $\tau = 5$ s and $\tau = 10$ s, obtaining for these $\kappa = 3.14$ and $\kappa = 3.17$, respectively, slightly smaller values than the kurtosis of $\kappa = 3.28$ found for $\tau = 1$ s.

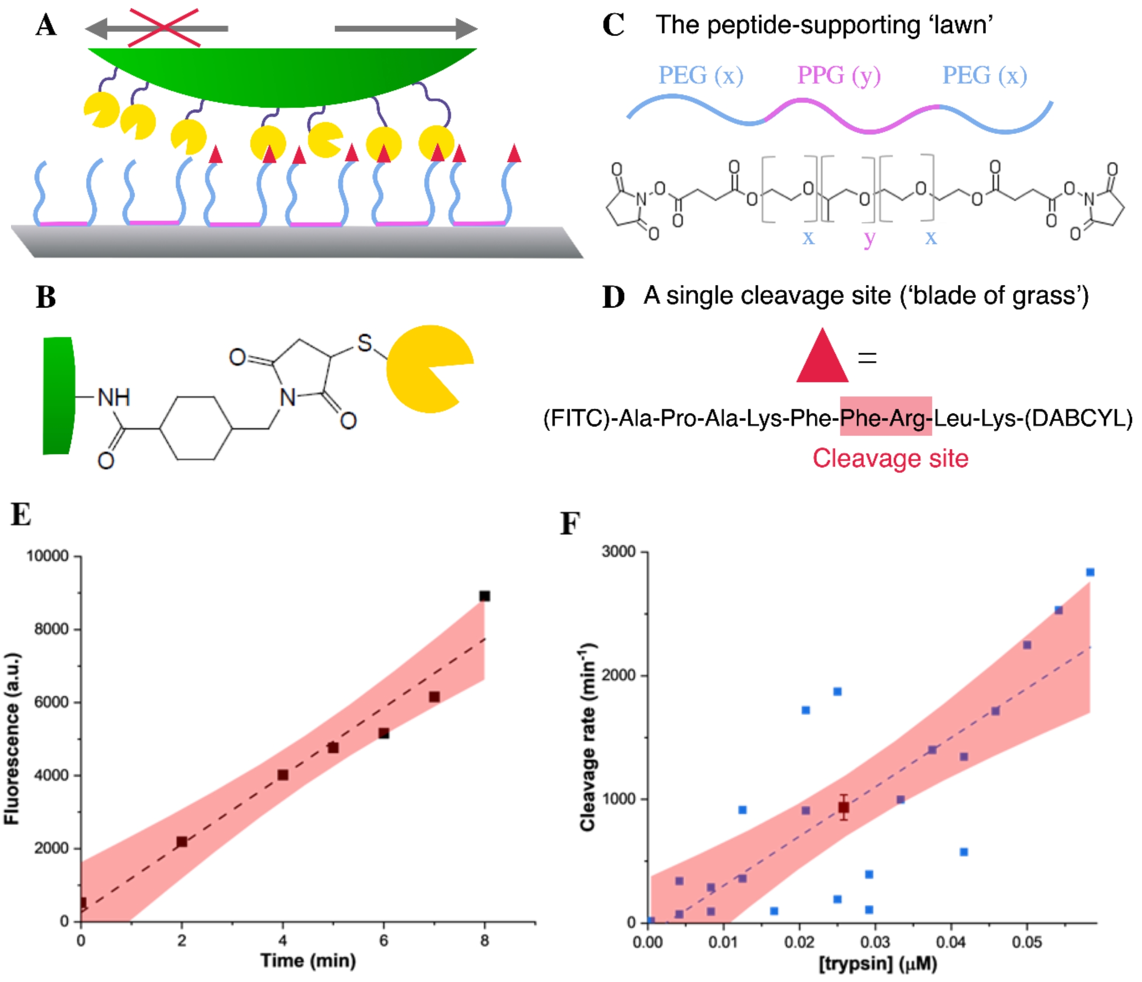

Supplementary Figure 1.
Details of Lawnmower design and construction. (A) The Lawnmower molecular motor concept is based on a burnt-bridge ratchet design. In the current design the LM consists of a central Dynabead hub (diameter: 2.8 µm) to which trypsin proteases are bound. (B) Each protease is linked to the hub via a heterobifunctional polymer linker (sulfo-SMCC). (C) The triblock copolymer lawn chemistry is used from ref. (*9*). Shown is a single NHS-F127 polymer, with NHS moieties on each of the terminal PEG groups. (D) A 'blade of grass' from the lawn is the peptide substrate, with the site of trypsin cleavage shown. The peptide is linked to the NHS-F127 via its central lysine sidechain. A fluorophore, fluorescein isothiocyanate (FITC), and quencher, 4-([4-(dimethylamino)phenyl]azo)benzoic acid (DABCYL), are located at the N and C termini of the peptide, respectively. (E) Fluorescence arising from cleavage of fluorogenic peptides by LMs at a concentration of $3 \cdot 10^7$ LMs/mL. Fluorescence increases linearly with time with rate 935 ± 100 min^{-1} (R^2=0.95). (F) Cleavage rates for different concentrations of trypsin (blue squares) and the same concentration of peptides as in (E). The cleavage rate scales linearly with trypsin concentration (R^2=0.62). The cleavage rate of LMs (dark red square) agrees with the cleavage rate of trypsin at $(2.6 \pm 0.6) \cdot 10^{-8}$ M within a 95% confidence band (pink). Significant dispersion of the reference measurements may be attributed to autocleavage of trypsin in bulk solution.

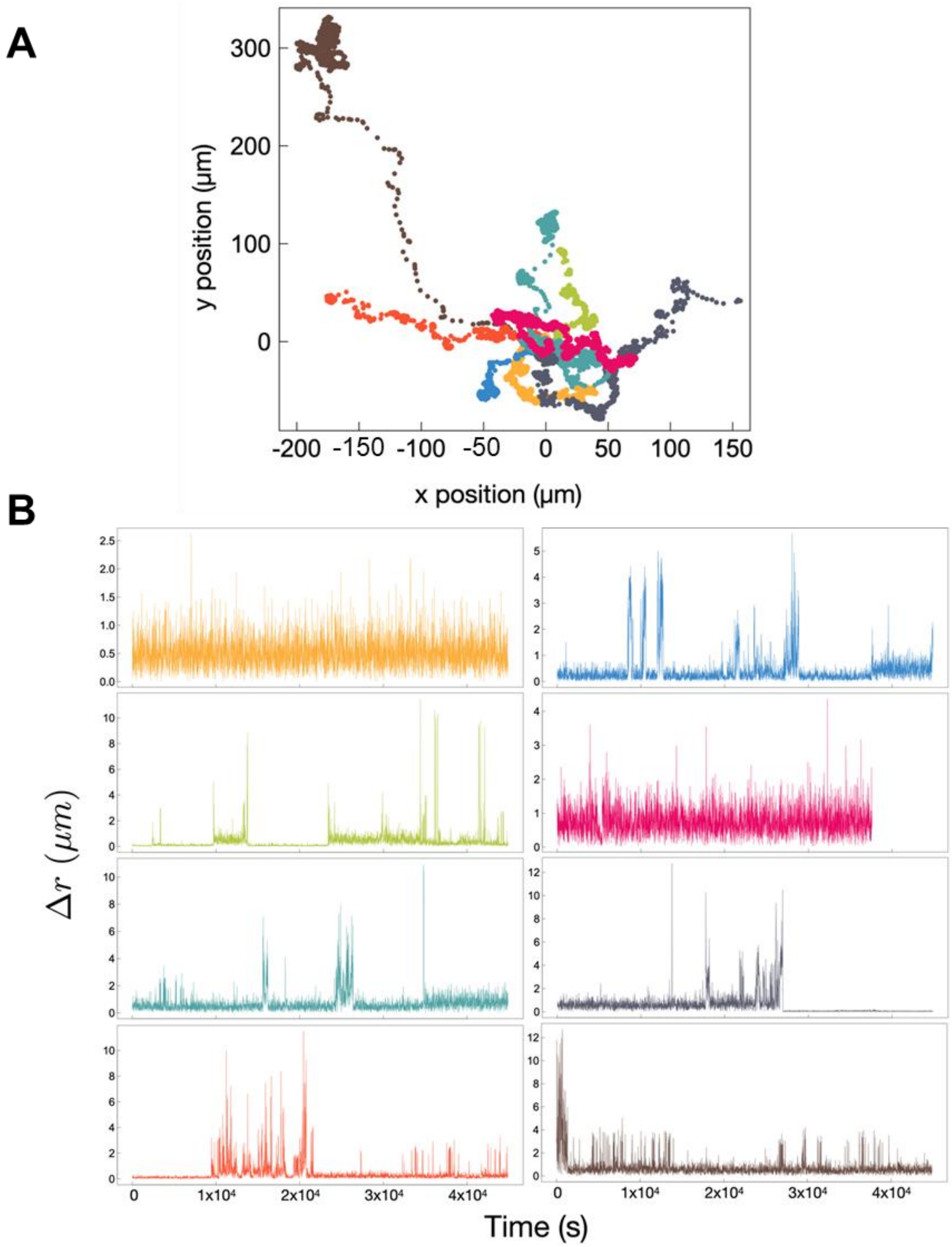

Supplementary Figure 2.

(A) Representative n = 8 LM trajectories on a peptide lawn from Fig. 1B, here shown over the entire 12.5-hour measurement time. **(B)** LM displacements vs time indicate highly heterogeneous dynamics. Displacements Δr are determined per 10-second time interval, for the exemplary LM trajectories shown in panel (A) and Fig. 1B, color-coded accordingly. Note: the vertical scale varies among panels.

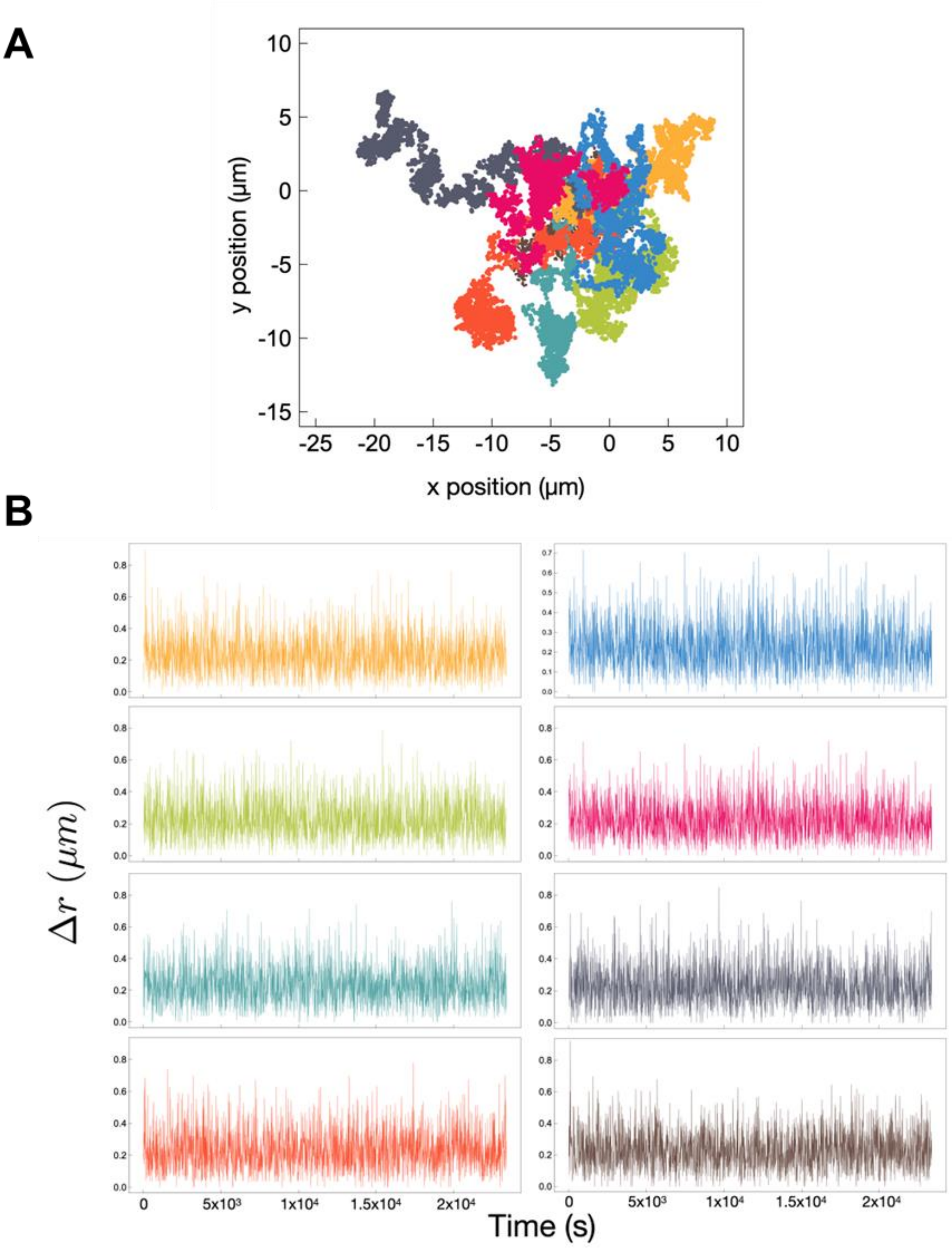

Supplementary Figure 3.

(A) Representative $n = 8$ LM trajectories on a bare lawn from Fig. 1B inset, here shown at a larger scale to more clearly show their diffusive nature. **(B)** LM displacements vs time indicate homogeneous dynamics, in stark contrast to dynamics on a peptide lawn (Supplementary Fig. 2). Displacements Δr are determined per 10-second time interval, for the exemplary LM trajectories shown in panel (A) and in the inset to Fig. 1B, color-coded accordingly. Note: the vertical scale varies slightly among panels.

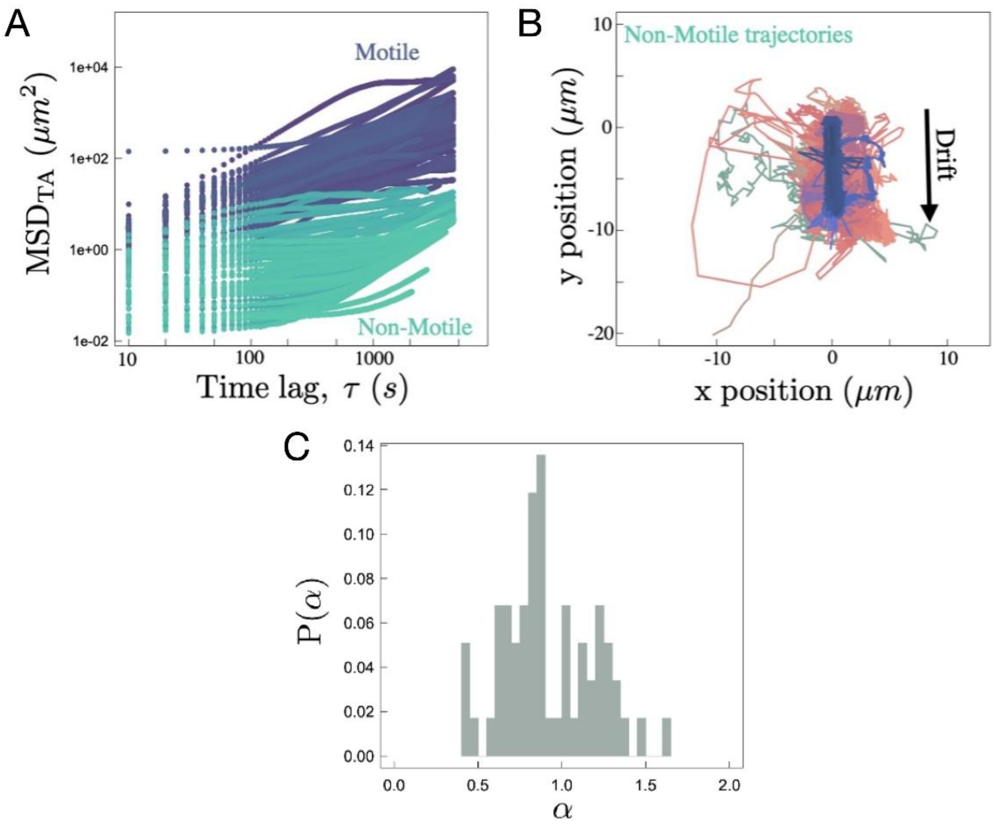

Supplementary Figure 4.

(A) MSD_{TA} of each LM on a peptide lawn in a single experiment. Trajectories are classified as motile or non-motile based on a threshold of MSD_{TA} = 10 μm^2 at τ = 4400 s (the longest time lag plotted and used for all MSD analysis of LMs on peptide lawns). (B) A plot of all non-motile trajectories in one 12.5 hr experiment (n = 55), centered at a common origin. Because of their extensive overlap, we concluded that the majority were irreversibly bound to the surface and thus used the average of these displacements as a stage drift trajectory. The drift trajectory was then subtracted from each motile LM's trajectory to correct for sample drift, prior to further analysis. Each non-motile trajectory is plotted with a unique color to help visually distinguish between trajectories. (C) Histogram of α_{TA} for each motile LM trajectory on a peptide lawn (n = 59). For each trajectory, α_{TA} was determined from a linear fit of its log(MSD_{TA}) vs log(τ) over all time lags from τ_{min} = 10 s to τ_{max} = 0.1 T_{msr}, representing 10% of the total measurement time of T_{msr} = 12.5 hours for the peptide lawn. Individual motile LMs on peptide lawns display a range of anomalous diffusive dynamics ranging from subdiffusive (α = 0.5) to superdiffusive (α = 1.4).

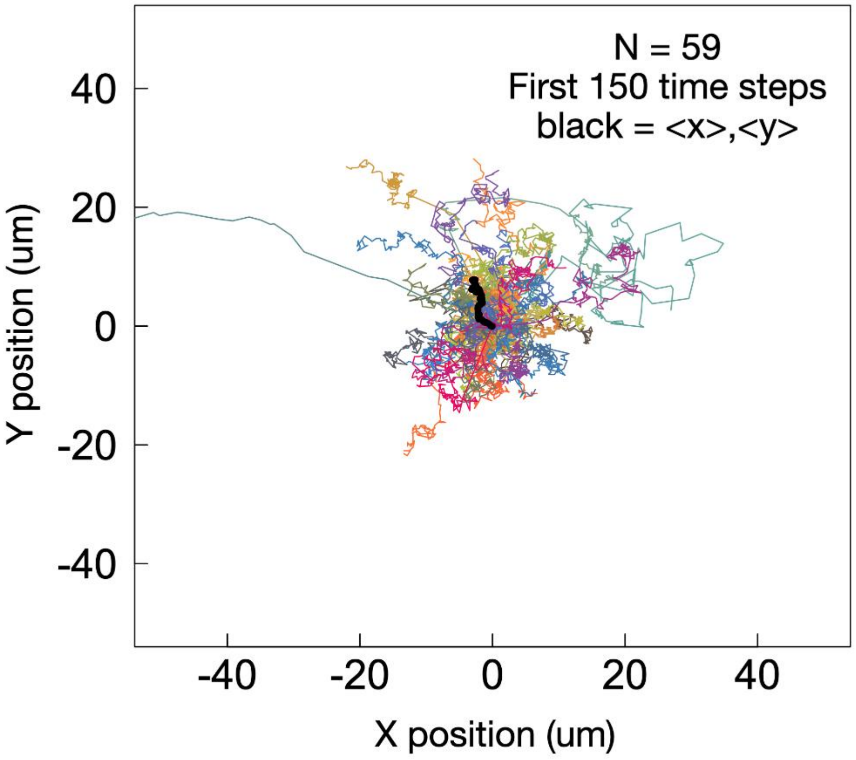

Supplementary Figure 5.

All *n*=59 motile Lawnmower trajectories over the first 1500 seconds when α_{EA} is strongly superdiffusive, displayed from a common origin. The superdiffusive dynamics do not arise from sample drift, as the trajectories visually appear isotropically distributed; the thick black line indicates the mean trajectory.

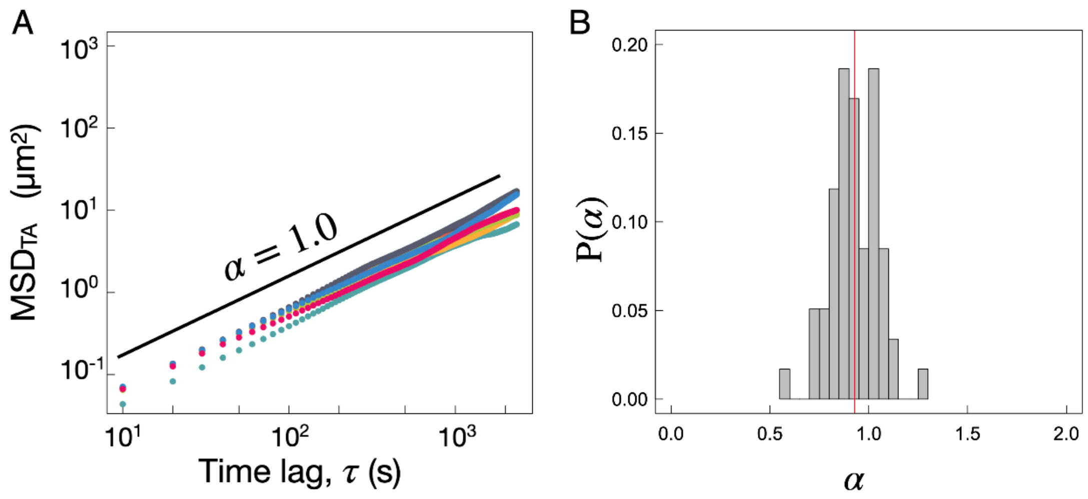

Supplementary Figure 6.

(A) The $\mathrm{MSD_{TA}}$ of all LMs on bare lawns throughout a single experiment of 6.25 hours. (B) Histogram of α_{TA} for each LM trajectory on a bare lawn (n = 59). For each trajectory, α_{TA} was determined from a linear fit of its log($\mathrm{MSD_{TA}}$) vs log(τ) over all time lags from τ_{min} = 10 s to τ_{max} = $0.1T_{\mathrm{msr}}$, representing 10% of the total measurement time of T_{msr} = 6.2 hours on the bare lawn. LMs on bare lawns exhibit α values clustered more closely around α = 1, exemplifying more conventionally diffusive dynamics.

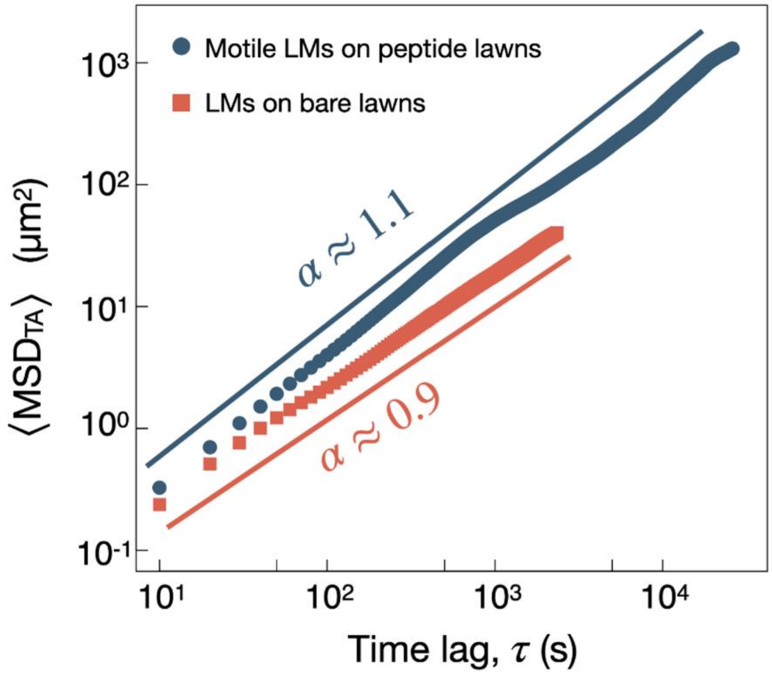

Supplementary Figure 7.

The ensemble-averaged MSD_{TA}, $<MSD_{TA}>$, on peptide lawns (*n*=59) and on bare lawns (*n*=59). Slightly superdiffusive dynamics are apparent throughout the 12.5-hour long trajectories of motile LMs on peptide lawns, while on bare lawns, LM motility is slightly subdiffusive.

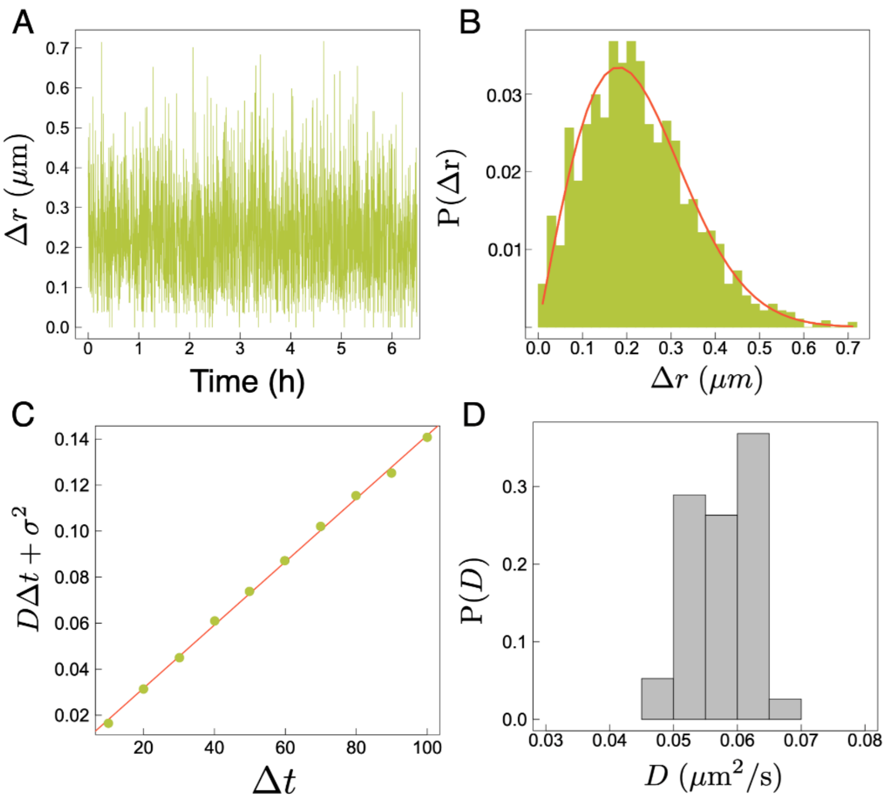

Supplementary Figure 8.

Lawnmower displacement distributions on a bare lawn are well described by conventional diffusion in 2D. (A) Representative plot of the time-dependent LM displacement Δr in each 10-second time interval throughout one trajectory (colored green in the inset to Fig. 1B and in Supplementary Fig. 3). (B) Probability distribution of displacements. Red line is a fit with the Rayleigh distribution describing conventional 2D diffusion (Eq. 1). (C) Fits to the displacement distributions at different Δt give estimates of $D\Delta t + \sigma_t^2$ where D is diffusion coefficient and σ_t^2 is tracking noise. For this trajectory, a linear fit provides $D = 0.051$ μm^2/s and $\sigma_t^2 = 0.0051$ μm^2. (D) Distribution of diffusion coefficients for all LMs on bare lawns, where D is determined for each trajectory as in panel (C). The results are tightly clustered, indicating homogeneous LM dynamics on bare F127 lawns. The average values across the ensemble are $\langle D \rangle = 0.056 \pm 0.004$ μm^2/s and $\langle \sigma_t^2 \rangle = 0.0024 \pm 0.0017$ μm^2 ($n = 59$; error represents standard deviation).

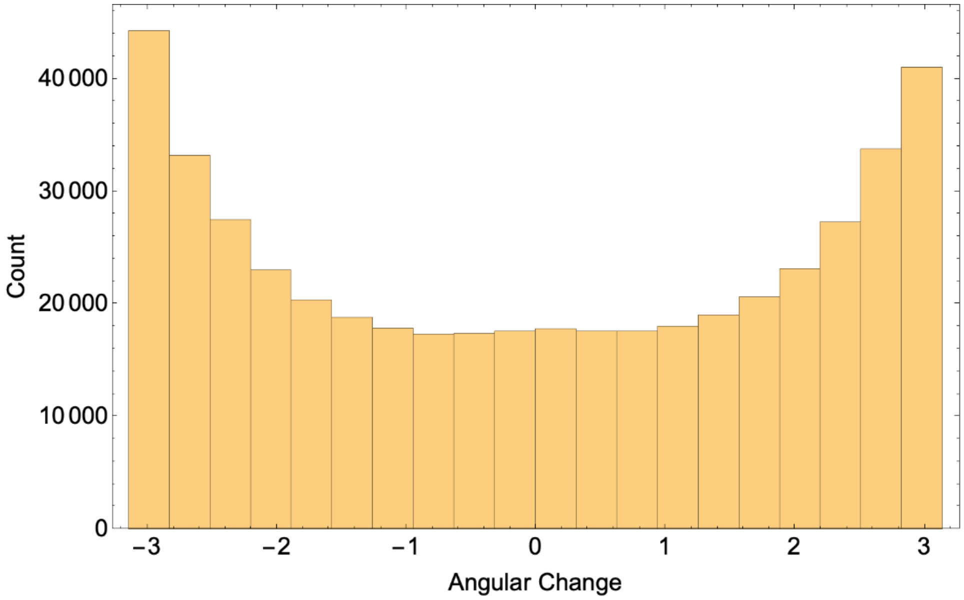

Supplementary Figure 9.

Distribution of angular change in direction between consecutive steps separated by Δt = 10 s for all LMs on peptide lawns, $\Delta\theta = (\Delta\theta_{i+1} - \Delta\theta_i)$, measured in radians. The peaks at ±π indicate that it is more likely for a LM to reverse its motion in consecutive intervals. This would be expected for an elastic restoring force associated with LM-peptide binding interactions.

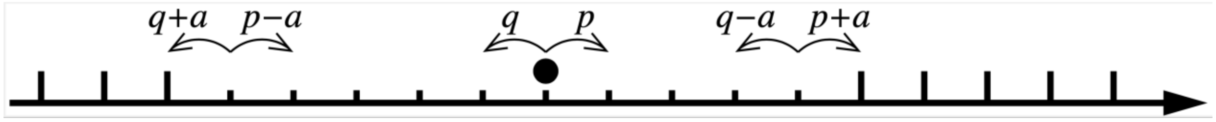

Supplementary Figure 10.

Simple model for the LM in 1D. The short vertical lines represent positions the LM has visited already, corresponding to an area of bare lawn. The long vertical lines represent unvisited positions with uncleaved lawn. In the region of bare lawn the LM is a usual random walker with probabilities q, p to jump to the left/right, respectively, with $p + q = 1$. For an unbiased random walker, $p = q = ½$; an overall net drift can be accounted for by setting $q = ½ - \delta$, $p = ½ +\delta$ ($-½ \leq \delta \leq 1/2$). At the edges of the bare region, stepping towards an uncleaved position is favored by the enzymatic action a ($0 \leq a \leq ½$), by adjusting q and p as indicated in the figure. If the LM moves to an uncleaved position, this position is cleaved and becomes part of the bare region.

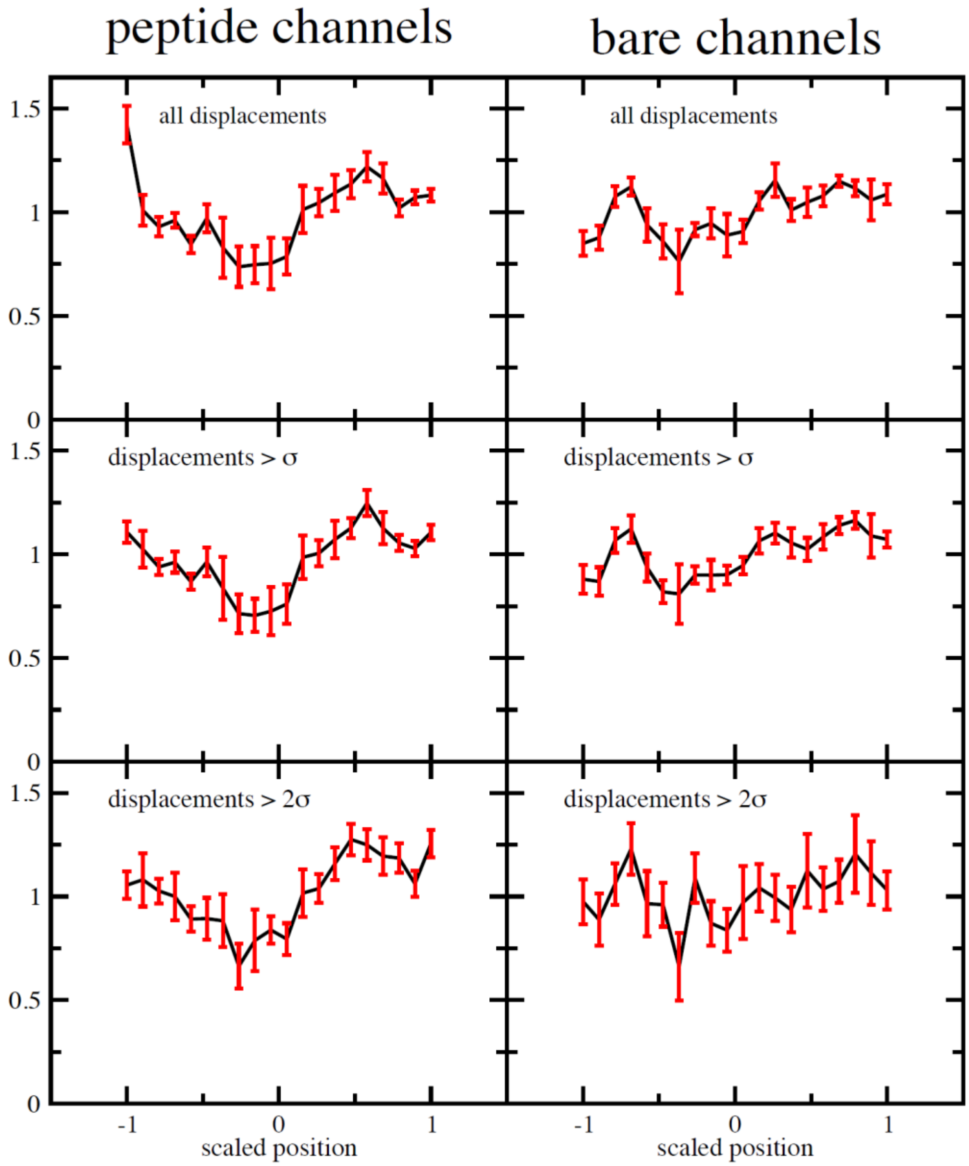

Supplementary Figure 11.

Probability ratio plots of experimentally measured displacements vs. diffusive displacements (black curves), as a function of scaled position (details in "Statistical analysis of LM displacements within previously visited regions" within Methods). The red error bars estimate the statistical uncertainty, and are obtained from the standard deviation among 5 randomly chosen cluster samples of the full data set. Left column: LM data. Right column: data from the control experiment with bare lawn. First row: all displacements are shown. Second row: only displacements larger than σ have been counted. Third row: only displacements larger than 2σ have been counted. The small left-right asymmetry visible in the data is probably due to an imperfect compensation of the background flow in the trajectory data (the net drift estimated from the average displacement per frame does not match the real background flow). The difference between the peptide and bare lawn channels is the pronounced dip in the black curves around the center (i.e. around scaled position 0), which is visible in all panels for the peptide

channels but not the bare channels. More precisely, the bare channel curves are consistent, within their statistical errors, with the hypothesis of a uniform probability ratio of 1 (horizontal line) in each panel, because the fluctuations along the curve for different scaled positions are comparable to the size of the error bars. In contrast, for the peptide channels the variations between the curves in the middle region (around scaled position 0) and at the edges (around scaled position ±1) are several error bar sizes (and, moreover, are more systematic than for the bare channels), making it extremely unlikely that the peptide channel data is statistically consistent with a uniform probability ratio.

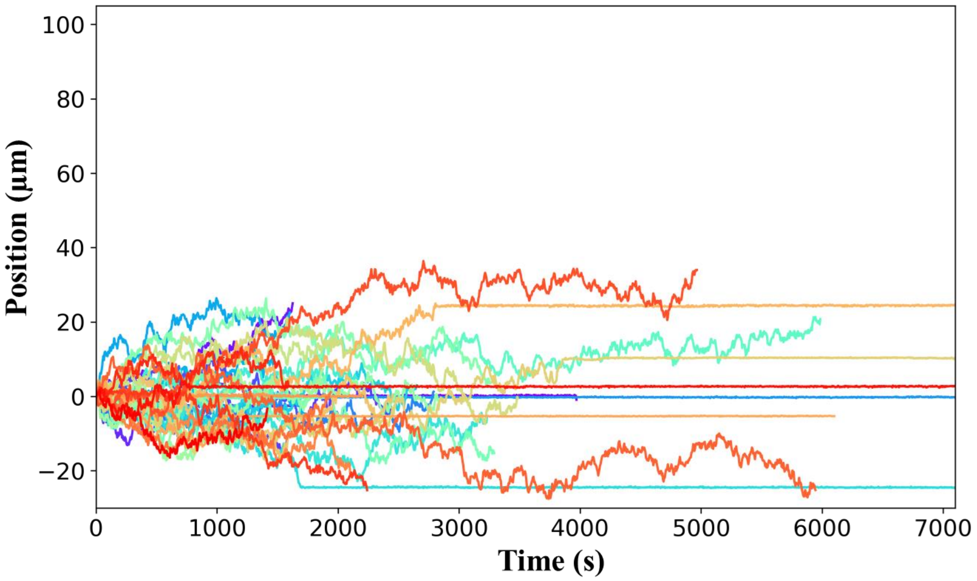

Supplementary Figure 12.

Trajectories from an independent replicate of LMs in peptide channels, in which some LMs show long periods of immotility. For this experiment, the kurtosis of the displacement distribution – excluding immotile portions of the trajectories – is $\kappa = 2.97$ at a time lag of $\tau = 5$ s (the sampling time) and $\kappa = 3.03$ at $\tau = 10$ s.

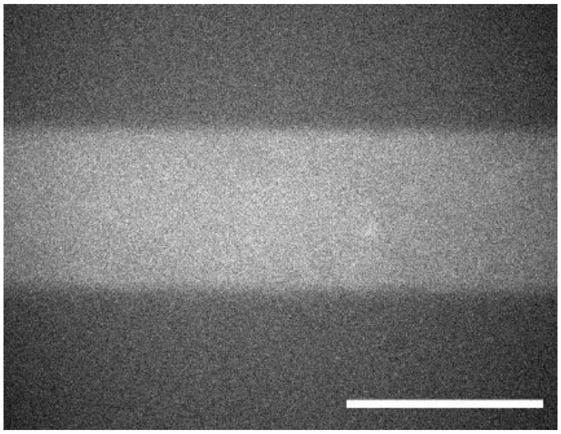

Supplementary Figure 13.

Selective deposition of the lawn is revealed by fluorescence imaging: cleaved peptide lawn is seen only in the channel, supported on a SiO_2 substrate. This experiment shows a wider channel than used for guiding LM motion. Scale bar 100 µm.

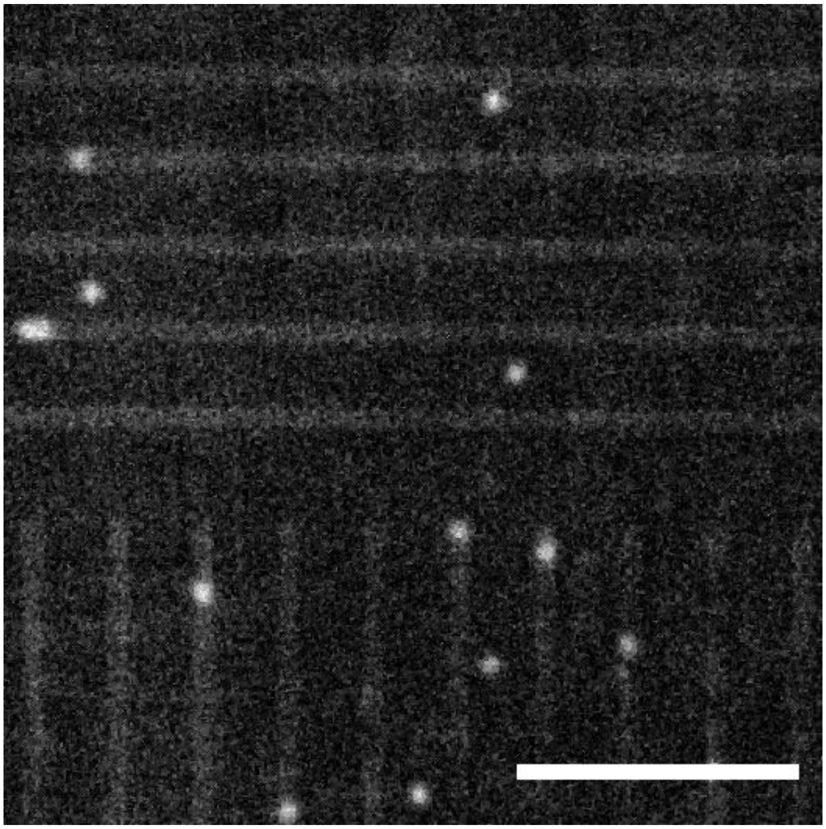

Supplementary Figure 14.

Fluorescence from cleaved peptides is selectively associated with channels of width 2.2 μm used in these LM experiments. Channel fluorescence was too faint to be detected after 1.5 hours of LM motion and also after addition of 50 μg/ml trypsin. Fluorescence was detectable after addition of 500 μg/ml trypsin, as shown here. Autofluorescence of the Dynabeads used in LM construction enables their visualization by fluorescence microscopy, seen as large dots in this image. Scale bar: 50 μm.

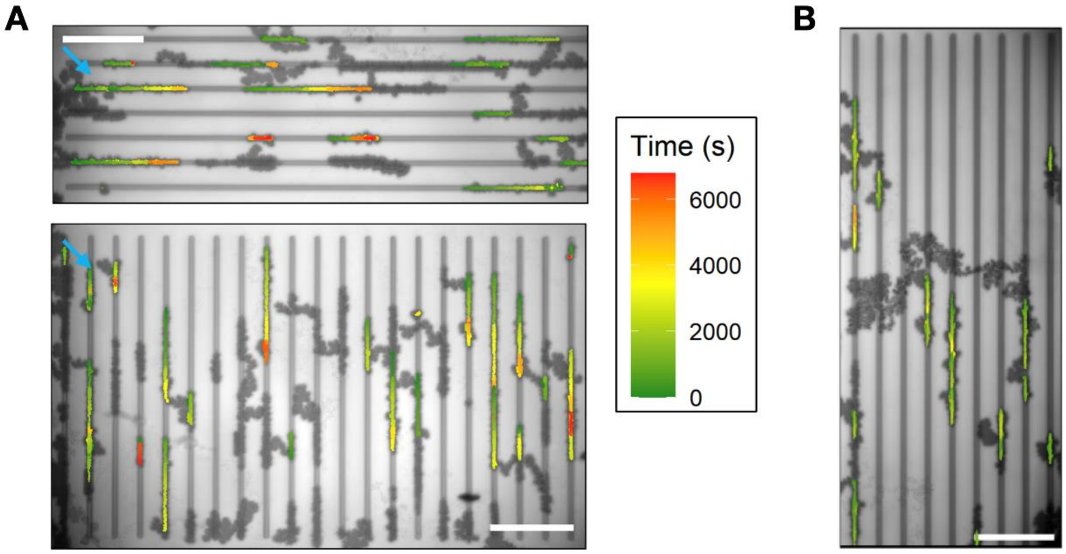

Supplementary Figure 15.

LMs and bare beads undergo occasional excursions out of their channels. (A) Overlay of all images in a LM channel experiment. LMs appear as dark circles in the brightfield images, and dark outlines show the paths of LMs (sometimes escaping from one channel and diffusing into another). Trajectories included in Fig. 2 of the manuscript and our analysis are color-coded by their duration. These remain confined within a single track throughout the trajectory, and do not collide with other particles or ends of the channels. Blue arrows indicate the direction of the background drift (horizontal 0.011 ± 0.002 mm/s, vertical 0.007 ± 0.001 μm/s). Scale bar is 50 μm. (B) Similar overlay and analyzed trajectories of unmodified beads on bare lawn channels. Here all the channels had the same orientation. Scale bar is 50 μm.

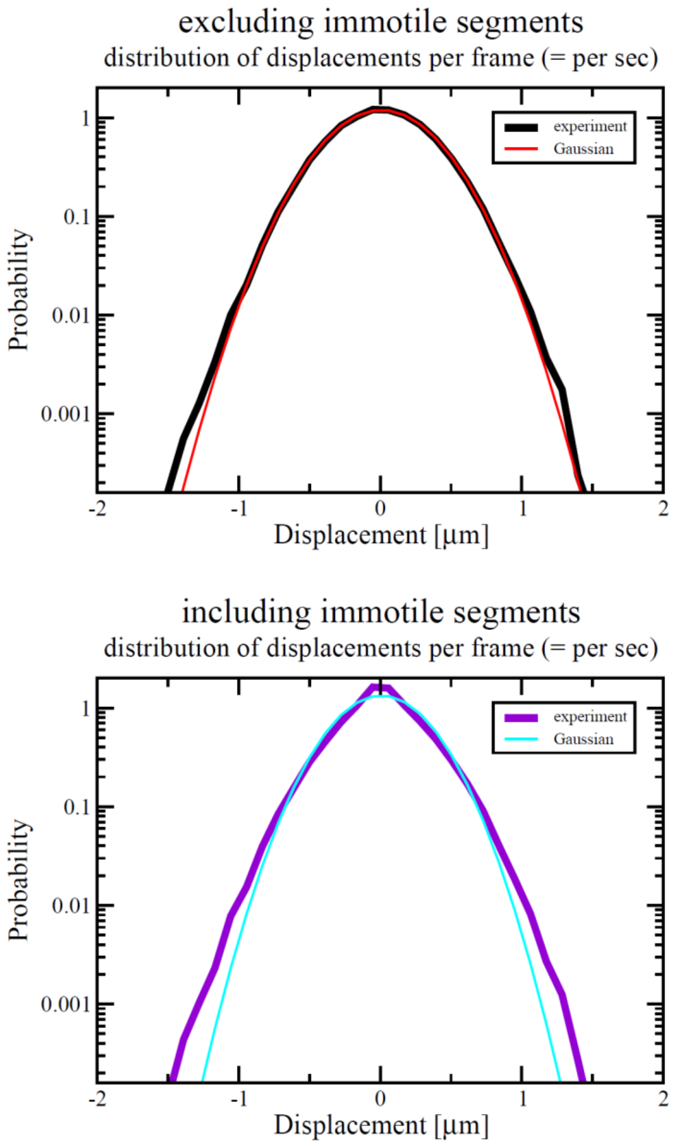

Supplementary Figure 16.

Comparison of displacement distributions of LMs in channels for τ = 1 second intervals, for trajectories truncated when they become immotile (top; same result as presented in Fig. 2F) and for entire trajectories (bottom). Immotile dwells contribute a narrow peak centered at $\Delta x = 0$. Consequently, the kurtosis of the distribution is increased and a Gaussian fit fails to capture the full width of the distribution. Distribution statistics are as follows. Top plot: mean displacement = 0.0086 µm, σ = 0.334 µm, κ = 3.28. Bottom plot: mean displacement = 0.0066 µm, σ = 0.298 µm, κ = 4.00. Gaussians are plotted using the mean and standard deviation of their respective distribution.

Supplementary References

1. S. Kovacic *et al.*, Design and Construction of the Lawnmower, An Artificial Burnt-Bridges Motor. *IEEE Transactions on NanoBioscience* **14**, 305 (2015). doi: 10.1109/TNB.2015.2393872
2. C. S. Korosec, Modelling and engineering artificial burnt-bridge ratchet molecular motors. PhD thesis, Simon Fraser University (2021).
3. I. N. Unksov *et al.*, Through the Eyes of Creators: Observing Artificial Molecular Motors. *ACS Nanoscience Au* **2**, 140 (2022).
4. L. Samii *et al.*, Time-dependent motor properties of multipedal molecular spiders. *Physical Review E* **84**, 031111 (2011).
5. C. S. Korosec, M. J. Zuckermann, N. R. Forde, Dimensionality-dependent crossover in motility of polyvalent burnt-bridges ratchets. *Physical Review E* **98**, 032114 (2018).
6. A. T. Blanchard, Burnt bridge ratchet motor force scales linearly with polyvalency: a computational study. *Soft Matter* **17**, 6056 (2021).
7. T. N. Akopian, A. F. Kisselev, A. L. Goldberg, Processive Degradation of Proteins and Other Catalytic Properties of the Proteasome from Thermoplasma acidophilum. *Journal of Biological Chemistry* **272**, 1791 (1997).
8. M. R. Nejadnik *et al.*, Adsorption of Pluronic F-127 on Surfaces with Different Hydrophobicities Probed by Quartz Crystal Microbalance with Dissipation. *Langmuir* **25**, 6245 (2009).
9. M. W. H. Kirkness, C. S. Korosec, N. R. Forde, Modified Pluronic F127 Surface for Bioconjugation and Blocking Nonspecific Adsorption of Microspheres and Biomacromolecules. *Langmuir* **34**, 13550 (2018).
10. A. Tanka-Salamon *et al.*, Structure and Function of Trypsin-Loaded Fibrinolytic Liposomes. *BioMed Research International* **2017**, 5130495 (2017).
11. M. Malmsten, K. Emoto, J. M. Van Alstine, Effect of Chain Density on Inhibition of Protein Adsorption by Poly(ethylene glycol) Based Coatings. *Journal of Colloid and Interface Science* **202**, 507 (1998).
12. Invitrogen, “Surface-activated Dynabeads” (2010).
13. Invitrogen, “Dynabeads® M-270 Amine product datasheet” (2012).
14. M. Matse, M. V. Chubynsky, J. Bechhoefer, Test of the diffusing-diffusivity mechanism using near-wall colloidal dynamics. *Physical Review E* **96**, 042604 (2017).
15. E. Schäffer, S. F. Nørrelykke, J. Howard, Surface Forces and Drag Coefficients of Microspheres near a Plane Surface Measured with Optical Tweezers. *Langmuir* **23**, 3654 (2007).
