# Supplementary material for: Motility of an autonomous protein-based artificial motor that operates via a burnt-bridge principle
Source: Nat Commun. 2024 Feb 23;15:1511. doi: 10.1038/s41467-024-45570-y (PMC10891099; doi:10.1038/s41467-024-45570-y)
Supplement: Supplementary file 3 — Description of Additional Supplementary Files [file 41467_2024_45570_MOESM3_ESM.pdf]

Description of Additional Supplementary Files

Supplementary Movie 1. Movie showing two Lawnmowers (2.8 µm diameter; black circles) on a 2D peptide lawn, one motile and the other immotile. Movie plays at 100X real time.

Supplementary Movie 2. Lawnmowers in peptide channels of two orthogonal orientations. Frame rate of the videos is 50 frames/s, actual imaging frame rate is 1 frame/s. Scale bar is 50 µm. Close-ups of selected trajectories enframed in yellow are in Movies 3 and 4.

Supplementary Movies 3, 4. Two selected Lawnmowers (corresponding regions in Movie 2 are marked in yellow) demonstrate prolonged immotile periods after which the motion continues. The LMs eventually jumped away from the channels, and further frames were cut off in the videos. Frame rate is 50 frames/s, scale bar is 15 µm.

Supplementary Movie 5. Particles in bare channels of one orientation. Frame rate of the videos is 50 frames/s. The actual imaging frame rate was 1 frame/s. Scale bar is 50 µm.
