# Supplementary material for: Motility of an autonomous protein-based artificial motor that operates via a burnt-bridge principle
Source: Nat Commun. 2024 Feb 23;15:1511. doi: 10.1038/s41467-024-45570-y (PMC10891099; doi:10.1038/s41467-024-45570-y)
Supplement: Supplementary file 9 — Reporting Summary [file 41467_2024_45570_MOESM9_ESM.pdf]

nature portfolio

Corresponding author(s): Korosec, Linke, Forde

Last updated by author(s): Jan. 20/24

Reporting Summary

Nature Portfolio wishes to improve the reproducibility of the work that we publish. This form provides structure for consistency and transparency in reporting. For further information on Nature Portfolio policies, see our Editorial Policies and the Editorial Policy Checklist.

Statistics

For all statistical analyses, confirm that the following items are present in the figure legend, table legend, main text, or Methods section.

n/a	Confirmed	
☐	☒	The exact sample size (*n*) for each experimental group/condition, given as a discrete number and unit of measurement
☐	☒	A statement on whether measurements were taken from distinct samples or whether the same sample was measured repeatedly
☐	☒	The statistical test(s) used AND whether they are one- or two-sided<br>*Only common tests should be described solely by name; describe more complex techniques in the Methods section.*
☒	☐	A description of all covariates tested
☐	☒	A description of any assumptions or corrections, such as tests of normality and adjustment for multiple comparisons
☐	☒	A full description of the statistical parameters including central tendency (e.g. means) or other basic estimates (e.g. regression coefficient) AND variation (e.g. standard deviation) or associated estimates of uncertainty (e.g. confidence intervals)
☒	☐	For null hypothesis testing, the test statistic (e.g. *F*, *t*, *r*) with confidence intervals, effect sizes, degrees of freedom and *P* value noted<br>*Give P values as exact values whenever suitable.*
☒	☐	For Bayesian analysis, information on the choice of priors and Markov chain Monte Carlo settings
☒	☐	For hierarchical and complex designs, identification of the appropriate level for tests and full reporting of outcomes
☒	☐	Estimates of effect sizes (e.g. Cohen's *d*, Pearson's *r*), indicating how they were calculated

Our web collection on statistics for biologists contains articles on many of the points above.

Software and code

Policy information about availability of computer code

Data collection	µManager
Data analysis	MTrack2 in ImageJ and FIJI ("Fiji is just imageJ") ; Two-dimensional LM experimental analysis: R (Version 4.2.3), Mathematica 13.1.0.0; One-dimensional LM experimental analysis: custom awk scripts, Python 3. Codes written to perform the analyses presented in this manuscript are available on GitHub (https://github.com/chapSKor/lawnmower).

For manuscripts utilizing custom algorithms or software that are central to the research but not yet described in published literature, software must be made available to editors and reviewers. We strongly encourage code deposition in a community repository (e.g. GitHub). See the Nature Portfolio guidelines for submitting code & software for further information.

Data

Policy information about availability of data

All manuscripts must include a data availability statement. This statement should provide the following information, where applicable:

- Accession codes, unique identifiers, or web links for publicly available datasets
- A description of any restrictions on data availability
- For clinical datasets or third party data, please ensure that the statement adheres to our policy

The datasets analysed during the current study are included within the accompanying TrajectoryData file.

Research involving human participants, their data, or biological material

Policy information about studies with human participants or human data. See also policy information about sex, gender (identity/presentation), and sexual orientation and race, ethnicity and racism.

Reporting on sex and gender	N/A
Reporting on race, ethnicity, or other socially relevant groupings	N/A
Population characteristics	N/A
Recruitment	N/A
Ethics oversight	N/A

Note that full information on the approval of the study protocol must also be provided in the manuscript.

Field-specific reporting

Please select the one below that is the best fit for your research. If you are not sure, read the appropriate sections before making your selection.

☒ Life sciences ☐ Behavioural & social sciences ☐ Ecological, evolutionary & environmental sciences

For a reference copy of the document with all sections, see nature.com/documents/nr-reporting-summary-flat.pdf

Life sciences study design

All studies must disclose on these points even when the disclosure is negative.

Sample size	Our experiments required hours-long tracking of the molecular motors in a single field of view of a microscope. Thus, sample sizes were based on how many motors it was possible to track without excessive overlaps of their trajectories.
Data exclusions	For one-dimensional analysis, we excluded motors and non-motor microspheres once they have left the channel tracks, or interacted with other objects (including the ends of a track). Furthermore, in that analysis we excluded data from an experiment where the sampling interval was longer (5 s) than in the reported data (1 s); data from that experiment are shown in Supplementary Figure 12.
Replication	One-dimensional results are obtained from observing the motors in tracks of two orthogonal orientations, providing a replicate of experiments within one chamber. Results from two separate chambers of peptide lawn channels are reported. Two-dimensional results are reported for one sample chamber. Previous measurements on a similarly prepared sample were performed (from which Movie 1 was clipped), but the primary data were lost in a computer failure. The similarity of dynamical responses in 2D and 1D measurements provides a cross-check of their validity.
Randomization	Not applicable as our data were not grouped.
Blinding	Not applicable as our data were labelled according to what experiment they came from, and analysis was performed by (or in close collaboration with) the people who did the experiments.

Reporting for specific materials, systems and methods

We require information from authors about some types of materials, experimental systems and methods used in many studies. Here, indicate whether each material, system or method listed is relevant to your study. If you are not sure if a list item applies to your research, read the appropriate section before selecting a response.

Materials & experimental systems

n/a	Involved in the study	
☒	☐	Antibodies
☒	☐	Eukaryotic cell lines
☒	☐	Palaeontology and archaeology
☒	☐	Animals and other organisms
☒	☐	Clinical data
☒	☐	Dual use research of concern
☒	☐	Plants

Methods

n/a	Involved in the study	
☒	☐	ChIP-seq
☒	☐	Flow cytometry
☒	☐	MRI-based neuroimaging
